# Deconstructing Multiorgan Sarcoidosis

**DOI:** 10.3390/jcm12062290

**Published:** 2023-03-15

**Authors:** Marc A. Judson

**Affiliations:** Division of Pulmonary and Critical Care Medicine, MC-91, Albany Medical College, Albany, NY 12208, USA; judsonm@amc.edu

Sarcoidosis is a multisystem granulomatous disease of unknown cause. Although this definition is commonplace throughout the medical literature, the meaning of the word “multisystem” is unclear. Many have assumed that “multisystem” is synonymous with “multiorgan.” Accordingly, some have required granulomatous inflammation in at least two organs for the diagnosis of sarcoidosis to be established [1].

However, in terms of clinical practice, multiple organ involvement is not required for the diagnosis of sarcoidosis. In the A Case Control Etiology of Sarcoidosis Study (ACCESS), half of the included cases (366/736) exhibited single-organ involvement [2]. The recent American Thoracic Society sarcoidosis diagnosis practice guidelines [3] mention certain forms of sarcoidosis organ involvement that are so specific for the diagnosis (e.g., lupus pernio) that evidence of additional organ involvement is not required. Criteria have also been developed to establish a clinical diagnosis of cardiac sarcoidosis without evidence of extracardiac disease (isolated cardiac sarcoidosis) [4]. The diagnosis of sarcoidosis usually requires a compatible clinical presentation, histologic evidence of non-caseating granulomatous inflammation, and exclusion of other disorders capable of producing similar histology or clinical features [3]. However, the diagnosis of sarcoidosis is never completely secure, because the diagnostic criteria of “a compatible clinical presentation” and “exclusion of other disorders capable of producing similar histology or clinical features” have not been clearly defined and are left to the arbitrary decision of the clinician [5,6]. Given this situation, single-organ involvement is adequate for the diagnosis of sarcoidosis provided that ample specific features for the diagnosis are also present [4,7], although admittedly this approach is arbitrary and not standardized. The requirement that two organs be involved to establish a diagnosis of sarcoidosis would increase the specificity of the diagnosis, but at the cost of a markedly diminished sensitivity.

Some “two organ purists” might argue that in the case that sarcoidosis is isolated to one organ, there is most likely occult involvement in a second organ that has escaped clinical detection [8]. It is known that biopsies of clinically uninvolved organs in sarcoidosis reveal granulomatous inflammation in 20 percent to more than 50 percent of cases [9,10,11]. However, I suspect that there are a sizable number of sarcoidosis patients with true isolated single-organ involvement. It is important to recognize that single-organ sarcoidosis may still be a systemic disease. Even when a single organ is involved with sarcoidosis, the disease often demonstrates systemic features including (a) anergy [12]; (b) parasarcoidosis syndromes where systemic symptoms develop from sarcoidosis that are not attributable to granulomatous deposition in a specific organ (e.g., small fiber neuropathy, pain, and fatigue syndromes [13]); and (c) the development of recurrent sarcoidosis in the allograft of sarcoidosis patients who undergo organ transplantation [14].

Understanding the development of multiorgan sarcoidosis may provide key insights into the immunopathogenesis of the disease. The immunopathogenesis of sarcoidosis is thought to involve entry of antigens into the host that are first identified and phagocytized by antigen-presenting cells, such as macrophages and dendritic cells [15]. Although there is significant evidence that forms of sarcoidosis may be autoimmune [16], the prevalent opinion is that antigens derived from exogenous sources are integrally involved in the granulomatous process [17]. These antigens are then processed by antigen-presenting cells and presented via human leukocyte antigen (HLA) class II molecules to a restricted set of T-cell receptors, primarily of the CD4+ class [18]. This activity induces polarization of the T cells to a T-helper-1 phenotype with subsequent cellular recruitment, proliferation, and differentiation leading to the sarcoid granuloma [15]. The two most common organs involved in isolated single-organ sarcoidosis are the lung and the skin [19]. Assuming sarcoidosis is instigated at least in part by exogenous antigens, this would suggest that the lung and skin are common “portals of entry” for the antigens involved in the development of sarcoidosis. This hypothesis is in keeping with the fact that the lung and the skin are particularly conductive sites of antigen capture [20] and adaptive immune responses [21].

Assuming that the initial sarcoidosis granuloma forms as a response to an exogenous antigen, the question arises as to how sarcoidosis granulomas develop in additional organs. It is possible that the causative antigens travel to other organs via the bloodstream or lymphatics. However, except possibly for Propionibacterium acnes [22], specific antigens have not been isolated within sarcoid granulomas. This suggests the possibility that sarcoid granulomas develop on the basis of autoimmunity in organs at or beyond the portal of entry site. It has been conjectured that that a foreign antigen itself or the initial granulomatous response at the portal of entry may lead to the exposure of self-peptides such that molecular mimicry occurs [23,24]. In this scenario, exposure of self-peptides promotes autoreactive T cells that can lead to the development of granulomatous inflammation to autoantigens in distant sites. Vimentin has been recognized as a possible autoantigen in sarcoidosis [25].

Possibly, the development on multiorgan sarcoidosis involves a two-hit hypothesis, wherein granulomatous inflammation develops in an isolated organ where the offending antigen is first encountered by the immune system. Development of multiorgan sarcoidosis may require a second process: the development of autoimmunity whereby granulomas can be formed in distant organs. 

Discovering the mechanisms responsible for multiorgan sarcoidosis is more than an academic exercise. Breakthroughs in this area may lead to prevention of dissemination of sarcoidosis into vital organs such as the heart and brain. To that end, the immunologic characteristics of isolated sarcoidosis versus multiorgan sarcoidosis should be rigorously explored.

## References

[1] Judson M.A., Baughman R.P. (2014). How many organs need to be involved to diagnose sarcoidosis?: An unanswered question that, hopefully, will become irrelevant. Sarcoidosis Vasc. Diffus. Lung Dis..

[2] Baughman R.P., Teirstein A.S., Judson M.A., Rossman M.D., Yeager H., Bresnitz E.A., DePalo L., Hunninghake G., Iannuzzi M.C., Johns C.J. (2001). Clinical Characteristics of Patients in a Case Control Study of Sarcoidosis. Am. J. Respir. Crit. Care Med..

[3] Crouser E.D., Maier L.A., Wilson K.C., Bonham C.A., Morgenthau A.S., Patterson K.C., Abston E., Bernstein R.C., Blankstein R., Chen E.S. (2020). Diagnosis and Detection of Sarcoidosis. An Official American Thoracic Society Clinical Practice Guideline. Am. J. Respir. Crit. Care Med..

[4] Terasaki F., Azuma A., Anzai T., Ishida Y., Isobe M., Inomata T., Ishibashi-Ueda H., Eishi Y., Kitakaze M., Kusano K. (2019). JCS 2016 Guideline on Diagnosis and Treatment of Cardiac Sarcoidosis—Digest Version. Circ. J..

[5] Judson M.A. (2018). The Diagnosis of Sarcoidosis: Attempting to Apply Rigor to Arbitrary and Circular Reasoning. Chest.

[6] Putman M., Patel J.J., Dua A. (2022). There is no diagnosis of exclusion in rheumatology. Rheumatology.

[7] Judson M.A. (2022). The time bomb of isolated cardiac sarcoidosis: Is it ticking?. Int. J. Cardiol..

[8] Birnie D.H., Nery P.B., Beanlands R.S. (2021). COUNTERPOINT: Should Isolated Cardiac Sarcoidosis Be Considered a Significant Manifestation of Sarcoidosis?. Chest.

[9] Chung Y.-M., Lin Y.-C., Huang D.-F., Hwang D.-K., Ho D.M. (2006). Conjunctival Biopsy in Sarcoidosis. J. Chin. Med. Assoc..

[10] Bilir M., Mert A., Ozaras R., Yanardag H., Karayel T., Senturk H., Tahan V., Ozbay G., Sonsuz A. (2000). Hepatic Sarcoidosis: Clinicopathologic Features in Thirty-seven Patients. J. Clin. Gastroenterol..

[11] Andonopoulos A.P., Papadimitriou C., Melachrinou M., Meimaris N., Vlahanastasi C., Bounas A., Georgiou P. (2001). Asymptomatic gastrocnemius muscle biopsy: An extremely sensitive and specific test in the pathologic confirmation of sarcoidosis presenting with hilar adenopathy. Ann. Rheum. Dis..

[12] Mathew S., Bauer K.L., Fischoeder A., Bhardwaj N., Oliver S.J. (2008). The Anergic State in Sarcoidosis Is Associated with Diminished Dendritic Cell Function. J. Immunol..

[13] Judson M.A., Baughman R.P., Valyere D.E. (2019). Parasarcoidosis syndromes. Sarcoidosis: A Clinician’s Guide.

[14] Ionescu D.N., Hunt J.L., Lomago D., Yousem S.A. (2005). Recurrent sarcoidosis in lung transplant allografts: Granulomas are of recipient origin. Diagn. Mol. Pathol..

[15] Judson M.A. (2019). A sarcoidosis clinician’s perspective of MHC functional elements outside the antigen binding site. Hum. Immunol..

[16] Rizzi L., Sabbà C., Suppressa P. (2022). Sarcoidosis and autoimmunity: In the depth of a complex relationship. Front. Med..

[17] Judson M.A. (2020). Environmental Risk Factors for Sarcoidosis. Front. Immunol..

[18] Baughman R.P., Culver D.A., Judson M.A. (2011). A concise review of pulmonary sarcoidosis. Am. J. Respir. Crit. Care Med..

[19] James W.E., Koutroumpakis E., Saha B., Nathani A., Saavedra L., Yucel R.M., Judson M.A. (2018). Clinical Features of Extrapulmonary Sarcoidosis Without Lung Involvement. Chest.

[20] Kurata A., Terado Y., Izumi M., Fujioka Y., Franke F.E. (2010). Where does the antigen of cutaneous sarcoidosis come from?. J. Cutan. Pathol..

[21] Bordignon M., Rottoli P., Agostini C., Alaibac M. (2011). Adaptive Immune Responses in Primary Cutaneous Sarcoidosis. J. Immunol. Res..

[22] Abe C., Iwai K., Mikami R., Hosoda Y. (1984). Frequent isolation of propionibacterium acnes from sarcoidosis lymph nodes. Zent. Bakteriol. Mikrobiol. Hyg. A.

[23] Zissel G., Muller-Quernheim J. (2016). Specific antigen(s) in sarcoidosis: A link to autoimmunity?. Eur. Respir. J..

[24] Häggmark A., Hamsten C., Wiklundh E., Lindskog C., Mattsson C., Andersson E., Lundberg I.E., Grönlund H., Schwenk J.M., Eklund A. (2015). Proteomic Profiling Reveals Autoimmune Targets in Sarcoidosis. Am. J. Respir. Crit. Care Med..

[25] Bagavant H., Cizio K., Araszkiewicz A.M., Papinska J.A., Garman L., Li C., Pezant N., Drake W.P., Montgomery C.G., Deshmukh U.S. (2022). Systemic immune response to vimentin and granuloma formation in a model of pulmonary sarcoidosis. J. Transl. Autoimmun..

